# Nonextensive Description of Charged-Particle Production in Ultrarelativistic Collisions

**DOI:** 10.3390/e28030298

**Published:** 2026-03-05

**Authors:** D. Rosales Herrera, J. C. Calderón Muñoz, J. R. Alvarado García, A. Fernández Téllez, J. E. Ramírez

**Affiliations:** 1 Facultad de Ciencias Físico Matemáticas, Benemérita Universidad Autónoma de Puebla, Apartado Postal 165, Puebla 72000, Puebla, Mexico; 2 Centro de Agroecología, Instituto de Ciencias, Benemérita Universidad Autónoma de Puebla, Apartado Postal 165, Puebla 72000, Puebla, Mexico

**Keywords:** Schwinger mechanism, string fragmentation, string tension fluctuations, temperature fluctuations, nonextensive statistical mechanics, *p*_T_ spectrum, heavy-tailed distributions

## Abstract

We study the production of charged particles in ultrarelativistic collisions by using the string fragmentation model. To do this, we describe the $p_{T}$ spectrum as the convolution of the Schwinger mechanism with string tension fluctuations that account for the stochastic nature of QCD. We found that heavy-tailed distributions are required to adequately reproduce the power-law tail of the $p_{T}$ spectrum experimentally observed. Additionally, the heavy-tailed characteristic is also necessary for the KNO scale invariance of intense color interactions modeling hard processes in this framework. In this way, the initial state admits a nonextensive picture, leading to a final state out of equilibrium, in which particle production occurs in small regions at different temperatures. Applying this framework to ALICE data, we observe trends in the power-law exponent as a function of event multiplicity and collision centrality. These trends are consistent with enhanced hard-particle production in small systems and with high-$p_{T}$-particle suppression in heavy-ion collisions.

## 1. Introduction

The study of particle production mechanisms in strong interactions at ultrarelativistic energies is fundamental to broadening our understanding of quantum chromodynamics (QCD), which involves the production of soft hadrons or hadrons redistributing their $p_{T}$ due to collective phenomena, for which standard perturbative techniques are not entirely practical. While perturbative QCD successfully describes hard processes and deep inelastic scattering at high-energy scales, it becomes unreliable at low-energy scales where the strong coupling constant takes large values, rendering the perturbative expansion divergent. Even high-order perturbative calculations accurately predict the hard component of the $p_{T}$ spectrum, but they do not capture the soft production mechanisms accurately.

Phenomenological models inspired by fundamental theoretical principles have been developed to describe soft QCD dynamics. For instance, the color string models describe the particle production resulting from the fragmentation of color flux tubes stretched between colliding partons. In these models, the produced particles acquire their transverse momentum through the Schwinger mechanism, originally formulated for vacuum pair creation in quantum electrodynamics [1], providing a framework for quark–antiquark production. As partons separate after an ultrarelativistic collision, the potential energy of their color interaction increases until a $q\bar{q}$ pair is spontaneously created. This new pair interacts with the previous pair via two new color fields, which can also produce $q\bar{q}$ pairs, unless the longitudinal kinetic energy of the created particles is not enough to produce new pairs. This process ultimately produces the observed final-state particles. Within this framework, quark confinement can be modeled using a linear potential, with the proportionality constant interpreted as string tension, which characterizes the strength of the confining color field. The characteristic Gaussian distribution modeling the $p_{T}$ distribution can be obtained through a semiclassical approximation to quantum tunneling through a linear potential [2]. This result establishes a direct connection between the energy threshold for the particle production rate and string tension as the central parameter for color string fragmentation, which remains constant for all parton interactions. This picture forms the foundation of the Lund string model [2], implemented in event generators such as PYTHIA [3], EPOS [4], and Herwig [5].

The fluctuating nature of the QCD vacuum and the stochastic dynamics of string formation can induce variations in the string tension parameter, which can be considered to be a random variable [6]. Then the $p_{T}$ spectrum must be obtained by convoluting the Schwinger mechanism with the probability distribution describing string tension fluctuations [7,8,9].

The consequences of a fluctuating string tension in the Schwinger mechanism were explored by Bialas in 1999 [6], who demonstrated that considering Gaussian string tension fluctuations yields a $p_{T}$ spectrum in the form of an exponential decay ($p_{T}$-exponential). The above derivation resembles the thermal model that has been used to describe experimental low-$p_{T}$ spectrum from the perspective of a hadron gas. Interestingly, this approach is similar to the energy fluctuations of the Boltzmann distribution in classical statistical mechanics [10]. Therefore, it is natural to identify the inverse of the decay constant as a temperature at which the particles are produced [11].

Experimental measurements, particularly at intermediate and high $p_{T}$, reveal deviations from simple exponential functions in the thermal description. The experimental $p_{T}$ spectrum exhibits a heavy tail that cannot be described using the thermal distribution, even at ISR energies [12,13,14,15]. These high-$p_{T}$ particles arise primarily from hard-partonic scattering, heavy-quark production, and hard-gluon emission processes, as implemented in the Lund model [2], requiring correspondingly higher string tensions during fragmentation. To generate such string tension values with sufficient probability, the distribution describing the string tensions must assign significantly more weight to the tail than a Gaussian distribution, motivating the use of heavy-tailed distributions [8]. This requirement is naturally fulfilled by the *q*-Gaussian distribution, which emerges from maximizing the Tsallis entropy in nonextensive statistical mechanics [16,17], describing systems with long-range correlations and fluctuating energy densities, features characteristic of QCD vacuum dynamics [9]. In 2023, Pajares and Ramírez showed that convoluting the Schwinger mechanism with *q*-Gaussian string tension fluctuations produces a confluent hypergeometric function *U* (or Tricomi function), successfully describing experimental data of the $p_{T}$ spectrum across the full $p_{T}$ range [8].

Importantly, *q*-exponential functions can be applied either to the initial state (via string tension fluctuations) or to the final state (directly modeling the $p_{T}$ distribution of observed particles). From the final-state perspective, the Tsallis *q*-exponential distribution captures both the exponential low-$p_{T}$ behavior and the power-law high-$p_{T}$ tail observed in experimental data. Phenomenologically, this function is equivalent to the quasi-power-law formula [18] introduced by Hagedorn [15] and others as a QCD-inspired parametrization [12,19], with both descriptions being related through a mapping between their parameters [20]. Remarkably, Wilk and Włodarczyk demonstrated that the Hagedorn function can also arise from the thermal approach convoluted with temperature fluctuations at kinetic freeze-out [21].

In this work, we discuss in detail the relationship between string tension fluctuations and temperature fluctuations in particle production at ultrarelativistic energies. We derive the distribution describing string tension fluctuation for models commonly used to describe experimental $p_{T}$ spectrum, including the Tricomi function, Hagedorn/Tsallis distributions, the two-component model [22], and the description beyond the string fragmentation [23]. For each model, we determine whether a corresponding temperature fluctuation distribution exists, thereby establishing the conditions under which the string tension and temperature fluctuation perspectives can be related, providing a way to interpret the physical origin of nonequilibrium features in particle production from phenomenological descriptions of the experimental $p_{T}$ spectrum. We analyze the asymptotic behavior of the $p_{T}$ spectrum at low and high $p_{T}$ and the corresponding string tension fluctuations at small and large string tension values. We identify the effective temperature characterizing the thermal regime and the power-law exponent that governs the nonextensivity of the initial state and nonthermal particle production.

The rest of the paper is organized as follows: In Section 2 we derive the Schwinger mechanism through a semiclassical approximation to quantum tunneling under a linear confining potential, establishing the connection to string fragmentation and hadronization in QCD. Section 3 is devoted to further discussing the framework of string tension fluctuations for the equilibrium case, where Gaussian fluctuations in string tension yield a thermal $p_{T}$ spectrum. In Section 4, we derive the string tension and temperature fluctuations for different models attempting to describe the entire $p_{T}$ spectrum, including the Tricomi function, Hagedorn/Tsallis distributions, the description beyond string fragmentation, and the two-component model. We establish the conditions under which the probability distribution corresponding to the temperature fluctuations exists and derive a general relationship between power-law tails in the $p_{T}$ spectrum and Pareto-type string tension fluctuations. Section 5 contains an analysis of several experimental datasets using the framework discussed in this manuscript. Our conclusions and discussion are presented in Section 6.

## 2. The Schwinger Mechanism and String Fragmentation in QCD

In the context of color interactions, a particle production mechanism in ultrarelativistic collisions can be derived from a semiclassical approach that considers a linear potential. Let us consider a particle of mass $m_{0}$ moving along the *ℓ*-direction with transverse momentum $p_{T}$, subject to the linear potential $V\left(\ell \right)=x^{2}\ell$, where $x^{2}$ is a constant related to the energy per unit length. The total relativistic energy is given by

(1) $$E=\sqrt{p_{\ell }^{2}+p_{T}^{2}+m_{0}^{2}}-x^{2}\ell ,$$

from which the longitudinal momentum $p_{\ell }$ is

(2) $$p_{\ell }^{2}\left(\ell \right)={\left(E+x^{2}\ell \right)}^{2}-m_{T}^{2},$$

where $m_{T}^{2}=m_{0}^{2}+p_{T}^{2}$ is the transverse mass and $p_{\ell }$ must be real-valued. Considering that the particle starts at ℓ=0, we require $E^{2}>m_{T}^{2}$, which determines the physically permitted regions where the particles move.

The classical *turning points* that delimit the classically accessible region occur when $p_{\ell }\left(\ell_{\pm }\right)=0$, giving

(3) $$\ell_{\pm }=\frac{-E\pm m_{T}}{x^{2}}.$$

The particle motion undergoes deceleration until it comes to rest at the turning points $\ell =\ell_{\pm }$, where the particle reverses direction, describing the characteristic “yoyo” motion.

The spacetime trajectory follows from Hamilton’s equations: the force equation $dp_{\ell }/dt=-\partial V/\partial \ell =-x^{2}$ and the relation $dE/dt=\left(dE/d\ell \right)\left(d\ell /dt\right)=-x^{2}\left(d\ell /dt\right)$. Integrating these relations yields a hyperbolic worldline of the form

(4) $$m_{T}^{2}=x^{4}\left[{\left(\ell -\ell_{0}\right)}^{2}-t^{2}\right],$$

where $\ell_{0}^{2}=m_{T}^{2}/x^{4}$. For massless particles ($m_{0}=0$), the motion occurs on the light cone, |ℓ|=|t|.

The yoyo model can describe the confinement of quark–antiquark pairs, considering they interact via a linear potential [2,24]. After scattering, the $q\bar{q}$ pair separates and decelerates until they reach their respective classical turning points, where they reverse direction and meet again, repeating the process indefinitely. However, in the context of quantum mechanics, the wave function does not vanish at these points but rather penetrates into the classically forbidden region [2]. This tunneling phenomenon leads to a finite probability of particle pair production, which can be calculated using the WKB approximation, as discussed in Section 2.

### Semiclassical Tunneling

The quantum description under the linear potential can be formulated through the Klein–Gordon equation with minimal coupling to a scalar potential [2,25], given by the coupling prescription $\partial_{\mu }\to \partial_{\mu }-ieA_{\mu }$ with the scalar potential $A_{0}=x^{2}\ell$ (and $\vec{A}=\vec{0}$), leading to

(5) $$\left[{\left(\partial_{t}-ix^{2}\ell \right)}^{2}-\partial_{\ell }^{2}-\nabla_{T}^{2}+m_{0}^{2}\right]\phi =0.$$

By substituting a solution of the form $\phi =\exp\left(-iEt\right)\exp\left(ip_{T}\cdot x_{T}\right)\psi \left(\ell \right)$ in Equation (5), we derive the following equation for ψ(ℓ):

(6) $$\psi^{\prime \prime }\left(\ell \right)+\left[{\left(x^{2}\ell +E\right)}^{2}-m_{T}^{2}\right]\psi \left(\ell \right)=0.$$

The above resembles the Schrödinger equation for a particle moving in the *ℓ*-direction with potential proportional to $-p_{\ell }^{2}$ and energy eigenvalues $-m_{T}^{2}/2m_{0}$. Following the Andersson treatment (see Ref. [2]), it is convenient to choose the energy origin such that E=0, which gives $p_{\ell }=i\sqrt{m_{T}^{2}-x^{4}\ell^{2}}$ (see Equation (2)), and the turning points are $\ell_{\pm }=\pm m_{T}/x^{2}$ (see Equation (3)). The momentum along the *ℓ*-direction ($p_{\ell }$) defines two regions with distinct descriptions: the classically allowed region where $p_{\ell }^{2}>0$ and the forbidden classical region where $p_{\ell }^{2}<0$. In the first case, the particles’ movement resembles the yoyo motion. On the other hand, there is only one way to observe an imaginary longitudinal momentum, which happens if the particles tunnel from the classically allowed region to the forbidden region. In the particular case of the linear potential, if two particles following the yoyo dynamics separate around the turning points, the system acquires sufficient energy for creating a new pair of particle–antiparticle that tunnels in the production points, which in the new local reference frame correspond to ℓ=0.

We use the WKB approximation to obtain the wave function describing the system in the forbidden classical region, giving the probability of the quantum tunneling. To apply this method to Equation (6), we must verify that the slowly varying potential condition is satisfied, corresponding to $|p_{\ell }^{\prime }\left(\ell \right)|\ll |p_{\ell }^{2}|$, or equivalently $|x^{4}\ell |\ll |p_{\ell }{|}^{3}$ [26]. This condition is fulfilled near ℓ=0, which is precisely the region of physical interest. In the WKB approximation, the probability for quantum tunneling through the classically forbidden region can be computed following the solution of the form ψ(ℓ)=exp(iW(ℓ)) [27]. By substituting in Equation (6), we obtain the following equation for *W*:

(7) $$i\frac{d^{2}W}{d\ell^{2}}-{\left(\frac{dW}{d\ell }\right)}^{2}+p_{\ell }^{2}=0.$$

By applying the slowly varying potential condition, W(ℓ) can be computed as

(8) $$W\left(\ell \right)=\int p_{\ell^{\prime }}\left(\ell^{\prime }\right)\;d\ell^{\prime }.$$

The wave function amplitude in the forbidden classical region is suppressed by the action integral S(ℓ) that corresponds to −iW(ℓ) [25,28]:

(9) $$S=\int_{0}^{\ell_{+}}\sqrt{m_{T}^{2}-x^{4}\ell^{2}}\;d\ell .$$

Introducing the change of variable $u=x^{2}\ell$ with $du=x^{2}d\ell$ and using the upper limit $x^{2}\ell_{+}=m_{T}$, the action becomes

(10) $$S=\frac{1}{x^{2}}\int_{0}^{m_{T}}\sqrt{m_{T}^{2}-u^{2}}\;du=\frac{\pi m_{T}^{2}}{4x^{2}},$$

where we have evaluated the integral as the area corresponding to a quarter-circle of radius $m_{T}$.

The pair production process involves the creation of both a particle and its antiparticle, each tunneling from the production point ℓ=0 toward opposite classical turning points. The WKB tunneling probability for each particle carries the standard suppression factor exp(−2S), from which the total pair production probability is $dw/dp_{T}^{2}\propto \exp\left(-4S\right)$. Thus, we derive the description of the $p_{T}$ distribution for particles produced by color string fragmentation, which is given by

(11) $$\frac{dN}{dp_{T}^{2}}\propto \mathrm{SM}\left(p_{T},x\right)\equiv \exp\left(-\frac{\pi p_{T}^{2}}{x^{2}}\right).$$

This result resembles the calculation by Schwinger in 1951 for electron–positron pair creation from the vacuum in the presence of a constant electric field E using the proper-time formalism in QED [1]. This mechanism is also similar to the Hawking–Unruh effect, in which the particle–antiparticle pairs are emitted near the event horizon of a black hole with a thermal spectrum [29,30].

In the context of color string fragmentation, the Schwinger mechanism describes the quantum tunneling process by which quark–antiquark pairs are spontaneously created from the QCD vacuum when the sum of the longitudinal energy ($p_{\ell }\left(0\right)$) of each interacting parton is sufficient for creating a new pair $q\bar{q}$ with masses $m_{q}$. Equation (11) represents the probability distribution for creating a new $q\bar{q}$ with transverse momentum $p_{T}$ through this tunneling process. Each newly created pair breaks the original color flux tube, forming two new color strings that can subsequently fragment through the same mechanism. This iterative process continues until the minimum energy condition is no longer satisfied. At this point, the final quark–antiquark combinations are identified as hadrons [25]. In each step, the pair creation is governed by the same tunneling mechanism, with the $p_{T}$ distribution of each newly produced pair given by Equation (11). The fragmentation cascade described above constitutes the foundation of the Lund string model [2]. Note that Equation (11) is a Gaussian distribution in $p_{T}$ centered at zero, where *x* characterizes the width of the distribution and has dimensions of energy as well as $p_{T}$. In subsequent sections, we will treat *x* as a random variable through which we introduce the stochastic nature of the QCD vacuum.

It is worth noting that the Schwinger mechanism (11) establishes the theoretical foundation for studying the $p_{T}$ spectrum from the perspective of QCD vacuum polarization. The string tension parameter can be used to describe experimental data from ISR, RHIC, and LHC, providing a means to validate nonperturbative QCD approaches through direct comparison with measurements.

## 3. Equilibrium Picture of Fluctuating String Tensions

String tension can be interpreted as the stored energy of QCD parton interactions within the color string fragmentation framework [24]. As a quantum field theory, the QCD vacuum exhibits intrinsic energy fluctuations. Consequently, the energy density of the color field is not necessarily constant across all partonic interactions. String tension fluctuations can incorporate not only the stochastic nature of QCD but also account for other effects that lead to variations in the strength of color interactions. For instance, the clustering of color strings and nonuniform parton spatial distributions, among others. In this way, the string tension fluctuations can be associated with the initial state of colliding systems.

In this section, we introduce a framework for incorporating string tension fluctuations into particle production phenomenology. The first case we examine concerns the fluctuations that give rise to the thermal description of the $p_{T}$ spectrum.

### Gaussian String Tension Fluctuations and the Thermal Spectrum

In 1999, Bialas [6] proposed that string tension fluctuations are described by a Gaussian distribution,

(12) $$P_{G}\left(x\right)=\sqrt{\frac{2}{\pi \varsigma^{2}}}\;\exp\left(-\frac{x^{2}}{2\varsigma^{2}}\right),$$

where x>0 measures the strength of the color field and ς is the standard deviation. In this picture, the $p_{T}$ spectrum arises from the convolution of the Schwinger mechanism with (12)

(13) $$\begin{array}{cc} \frac{dN}{dp_{T}^{2}}\; & \propto \int_{0}^{\infty }P_{G}\left(x\right)\;\mathrm{SM}\left(p_{T},x\right)\;dx\\ \; & =\sqrt{\frac{2}{\pi \varsigma^{2}}}\int_{0}^{\infty }\exp\left(-\frac{x^{2}}{2\varsigma^{2}}\right)\;\exp\left(-\pi p_{T}^{2}/x^{2}\right)\;dx. \end{array}$$

Integral (13) can be evaluated using the identity [6]

(14) $$\exp\left(-u/\sqrt{s}\right)=\int_{0}^{\infty }\exp\left(-st\right)\;\frac{u}{2\sqrt{\pi t^{3}}}\;\exp\left(-u^{2}/4t\right)\;dt,$$

which leads to the thermal spectrum,

(15) $$\frac{dN}{dp_{T}^{2}}\propto \exp\left(-p_{T}/T\right),$$

where the effective temperature parameter

(16) $$T=\sqrt{\frac{\varsigma^{2}}{2\pi }}$$

is identified as the inverse of the slope of the exponential decay, in close analogy with the Boltzmann distribution [11,31,32]. This parameter is associated with the soft scale characterizing low-$p_{T}$-particle production and quantifies the flattening of the soft region of the $p_{T}$ spectrum [33]. This temperature definition will play a central role in the rest of the manuscript for models that recover the $p_{T}$-exponential decay; in some cases, the effective temperature can capture information from regions beyond the low-$p_{T}$ regime, serving as a standard parameter for comparing different models.

**Figure 1:**
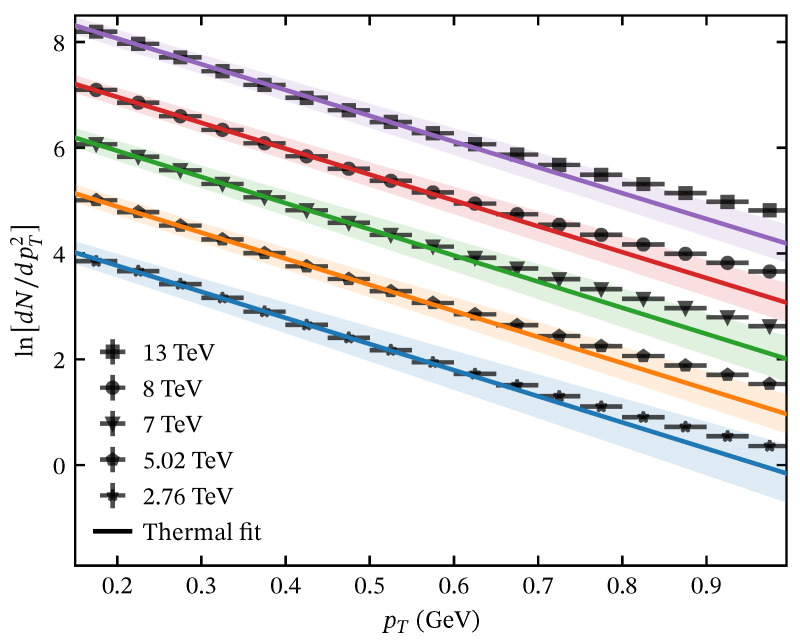
Thermal fits in the optimal $p_{T}$ range, determined by the minimization of $\chi^{2}/$NDF, showing the region where $\ln(dN/dp_{T}^{2})$ behaves linearly as a function of $p_{T}$. The shaded regions correspond to uncertainty propagation.

The thermal distribution is a well-established model that has been used to describe experimental data at low $p_{T}$ [12,15,32]. However, the fit quality deteriorates when it is extended to a wider $p_{T}$ range: $\chi^{2}/\mathrm{NDF}$ increases significantly as more high-$p_{T}$ points are included. To quantify this effect, an upper bound $p_{T}^{\max}$ can be determined for the $p_{T}$ range where there is a minimum $\chi^{2}/$NDF providing an acceptable fit quality [20]. In Figure 1, we show the optimized fits to experimental data of charged particles produced in minimum-bias pp collisions reported by the ALICE Collaboration [34]. We show the plot of $\ln(dN/dp_{T}^{2})$ as a function of $p_{T}$, exhibiting the region where the $p_{T}$ spectrum behaves as a $p_{T}$-exponential decay, which corresponds to $\ln(dN/dp_{T}^{2})$ behaving as a linear function of $p_{T}$. Note that the linear behavior starts to deviate around $p_{T}∼0.7\;\mathrm{GeV}$, indicating a clear departure from the thermal behavior.

It is noteworthy that the extracted temperature parameter *T* from these fits saturates at approximately 0.20GeV across the range of minimum-bias pp collision energies, as we further discuss in Section 5. This value is remarkably close to the weighted average mass of hadrons produced at low $p_{T}$, approximately $\langle m\rangle \approx w_{\pi }m_{\pi }+w_{K}m_{K}+w_{p}m_{p}+\cdots \approx 0.2\;\mathrm{GeV}$, where the weights $w_{k}$ reflect the measured abundances of hadrons with mass $m_{k}$ [35]. This suggests that the soft scale *T* for the thermal distribution encodes the typical mass scale of the hadrons that dominate the low-$p_{T}$ spectrum.

## 4. Nonextensive Fluctuations

Experimentally, the probability of producing high-$p_{T}$ particles is not negligible. In fact, a heavy tail in the $p_{T}$ spectrum has been observed in the experimental data not only at LHC energies, but also at ISR energies [12,13,14,15,32]. From the viewpoint of fluctuating color string tensions, a Gaussian distribution does not assign enough probability to large *x* values, which are needed to produce high-$p_{T}$ particles. A natural generalization of the Gaussian string tension fluctuations was introduced by Pajares and Ramírez [8], who proposed a heavy-tailed *q*-Gaussian distribution for *x*. Within this framework, the probability of large string tensions is enhanced, allowing for the emission of hard partons that give rise to high-$p_{T}$ hadrons, consistent with the picture of string fragmentation in the Lund model [2].

The *q*-Gaussian distribution is given by

(17) $$P_{qG}\left(x\right)=\sqrt{\frac{2(q-1)}{\pi }}\frac{\Gamma \left(\frac{1}{q-1}\right)}{\sigma \Gamma \left(\frac{1}{q-1}-\frac{1}{2}\right)}{\left[1+\frac{\left(q-1\right)x^{2}}{2\sigma^{2}}\right]}^{\frac{1}{1-q}},$$

where *q* is the nonextensive parameter taking values less than three to assure proper normalization and σ is a scale parameter measuring the width of the distribution. Notably, this function recovers the Gaussian distribution (12) in the limit q→1.

The asymptotic behaviors of (17) are

(18) $$P_{qG}\left(x\right)\propto \left\{\begin{array}{cc} 1-\frac{x^{2}}{2\sigma^{2}}+O\left(x^{4}\right),\; & x\to 0,\\ x^{\frac{2}{1-q}},\; & x\to \infty . \end{array}\right.$$

The series expansion at the second order for low string tension values approximates to $\exp(-x^{2}/2\sigma^{2})$, which corresponds to the Gaussian string tension fluctuations. At large string tension values, the *q*-Gaussian distribution follows a power law. This reveals that (17) is a heavy-tailed distribution.

Considering the *q*-Gaussian string tension fluctuations within the Schwinger mechanism, we obtain

(19) $$\begin{array}{cc} \frac{dN}{dp_{T}^{2}}\; & \propto \int_{0}^{\infty }P_{qG}\left(x\right)\;\mathrm{SM}\left(p_{T},x\right)\;dx\\ \; & =\sqrt{\frac{2(q-1)}{\pi }}\frac{\Gamma \left(\frac{1}{q-1}\right)}{\sigma \Gamma \left(\frac{1}{q-1}-\frac{1}{2}\right)}\int_{0}^{\infty }{\left[1+\frac{\left(q-1\right)x^{2}}{2\sigma^{2}}\right]}^{\frac{1}{1-q}}\exp\left(-\pi p_{T}^{2}/x^{2}\right)\;dx. \end{array}$$

To evaluate and simplify this integral, a change of variables is considered, i.e.,

(20) $$a_{q}=\frac{1}{q-1}-\frac{1}{2},\;\tau =\frac{\left(q-1\right)x^{2}}{2\sigma^{2}},$$

so that $dx=\sigma \;\tau^{-1/2}\;d\tau /\sqrt{2(q-1)}$. Substituting the above into (19) gives [8]

(21) $$\begin{array}{cc} \frac{dN}{dp_{T}^{2}}\; & \propto \frac{\Gamma \left(\frac{1}{q-1}\right)}{\sqrt{\pi }\;\Gamma \left(a_{q}\right)}\int_{0}^{\infty }\exp\left(-\frac{\pi p_{T}^{2}\left(q-1\right)}{2\sigma^{2}}\tau \right)\tau^{a_{q}-1}{\left(1+\tau \right)}^{-a_{q}-\frac{1}{2}}\;d\tau . \end{array}$$

Equation (21) has the form of the Tricomi function U(a,b,z), which is defined by

(22) $$U\left(a,b,z\right)=\frac{1}{\Gamma (a)}\int_{0}^{\infty }\exp\left(-zt\right)\;t^{a-1}{\left(1+t\right)}^{b-a-1}\;dt.$$

By matching parameters, the spectrum becomes

(23) $$\frac{dN}{dp_{T}^{2}}\propto W_{U}=\frac{1}{\sqrt{\pi }}\;\Gamma \;\left(\frac{1}{q-1}\right)U\;\left(\frac{1}{q-1}-\frac{1}{2},\;\frac{1}{2},\;\frac{\pi p_{T}^{2}\left(q-1\right)}{2\sigma^{2}}\right),$$

which requires q<3/2 for assuring the convergence of the average $p_{T}$.

The asymptotic behaviors of the *U* function are crucial to understanding the resulting $p_{T}$ spectrum in both the low- and high-$p_{T}$ regimes. For the special case b=1/2, which appears throughout our analysis, we have:

(24) $$U\left(a,\frac{1}{2},z_{0}p_{T}^{2}\right)\propto \left\{\begin{array}{cc} \frac{1}{\Gamma (a+\frac{1}{2})}-\frac{2\sqrt{z_{0}}p_{T}}{\Gamma (a)}\;+\frac{2az_{0}p_{T}^{2}}{\Gamma (a+\frac{1}{2})}\;+O\left(p_{T}^{3}\right),\; & p_{T}\to 0,\\ p_{T}^{-2a}\left(1-a\left(a+\frac{1}{2}\right)z_{0}^{-1}p_{T}^{-2}+O\left(p_{T}^{-4}\right)\right),\; & p_{T}\to \infty . \end{array}\right.$$

The low-$p_{T}$ expansion contains terms that give rise to an exponential behavior in $p_{T}$, while the high-$p_{T}$ limit yields a power-law tail.

Using the asymptotic expansion (24) with $a=\frac{1}{q-1}-\frac{1}{2}$ and $z_{0}=\frac{\pi (q-1)}{2\sigma^{2}}$, we identify that at low $p_{T}$, the $p_{T}$ spectrum behaves according to

(25) $$W_{U}\propto \exp\left(-p_{T}/T_{U}\right),$$

where the effective temperature is

(26) $$T_{U}=\sigma \frac{\Gamma \left(\frac{1}{q-1}-\frac{1}{2}\right)}{\sqrt{2\pi (q-1)}\;\Gamma \left(\frac{1}{q-1}\right)}.$$

At high $p_{T}$, the power-law behavior of *U* gives

(27) $$W_{U}\propto {\left(p_{T}^{2}\right)}^{-a_{q}}={\left(p_{T}^{2}\right)}^{\frac{1}{1-q}+\frac{1}{2}},$$

as discussed in Ref. [8].

Both asymptotic limits, the thermal behavior at low $p_{T}$ and the power law describing the heavy tail of the $p_{T}$ spectrum, are important for describing the experimental data, as shown in previous analyses [8,9,20,36]. These characteristics will appear repeatedly in the models discussed below. Additionally, we have the proper definition of the temperature for the *U* function (23), which smoothly approaches the thermal temperature in the limit q→1. On the other hand, the power-law behavior at high $p_{T}$ represents a significant deviation from the thermal description as $p_{T}$ increases.

As we further discuss in the following sections, the nonextensivity encoded in string tension fluctuations describes a system out of thermal equilibrium, and it is possible to determine the temperature fluctuations for some of these approaches.

### 4.1. Hagedorn Distribution and Temperature Fluctuations

The Hagedorn function is a widely used QCD-motivated parametrization of the $p_{T}$ spectrum, given by [15]

(28) $$\frac{dN}{dp_{T}^{2}}\propto {\left(1+\frac{p_{T}}{p_{0}}\right)}^{-m},$$

where $p_{0}$ sets an effective $p_{T}$ scale controlling the transition from the soft regime to the hard scatterings and *m* is the power-law exponent [15]. Note that by choosing the appropriate change of variables, the Hagedorn function can adopt the form of a *q*-exponential distribution. By setting $p_{0}=mT_{\mathrm{Hag}}$ in Equation (28) and taking $m=1/(q_{e}-1)$ or alternative parametrizations, the Hagedorn function encompasses different versions of the Tsallis distribution frequently used to fit the experimental $p_{T}$ spectrum [37,38,39].

The Hagedorn function correctly captures the asymptotic behaviors of experimental data. At low $p_{T}$ values, Equation (28) behaves as an exponential decay $\exp(-p_{T}/T_{\mathrm{Hag}})$ with soft scale

(29) $$T_{\mathrm{Hag}}=\frac{p_{0}}{m}.$$

At high $p_{T}$ values, it decreases as a power law, $dN/dp_{T}^{2}∼p_{T}^{-m}$ [15].

Remarkably, the Hagedorn function can be rewritten as a convolution of the thermal distribution (15) with temperature fluctuations [21],

(30) $${\left(1+\frac{p_{T}}{p_{0}}\right)}^{-m}=\int_{0}^{\infty }\exp\left(-p_{T}/T\right)\;\frac{\Gamma (1/T;m,p_{0})}{T^{2}}\;dT,$$

where $\Gamma \left(s;a,b\right)=b^{a}s^{a-1}\exp\left(-bs\right)/\Gamma \left(a\right)$ is the Gamma distribution (see Appendix A for a detailed computation). Equation (30) shows that the Hagedorn function emerges from temperature fluctuations described by

(31) $$T_{\mathrm{Hag}}\left(T\right)=\frac{\Gamma (1/T;m,p_{0})}{T^{2}}=\frac{p_{0}^{m}}{\Gamma (m)}\;T^{-(m+1)}\;\exp\left(-p_{0}/T\right),$$

which exhibits the variations in temperature in small regions of the system where particles are created.

In thermodynamics, temperature fluctuations indicate that the system departs from thermal equilibrium, characterized by a single and well-defined temperature *T* that is uniform throughout the system [10,31]. The analogy of thermal equilibrium for particle production in ultrarelativistic collisions follows the thermal distribution discussed in Section 3, which can be expressed as

(32) $$\exp\left(-p_{T}/T\right)=\int_{0}^{\infty }\exp\left(-p_{T}/\tau \right)\delta \left(\tau -T\right)d\tau ,$$

where δ(x) is the Dirac delta function.

### 4.2. String Tension Fluctuations of the Hagedorn Function

Let us further discuss the implications of the nonextensive properties of the system where particles are produced from the perspective of the Hagedorn function.

We recall that the thermal distribution (15) is obtained by convoluting the Schwinger mechanism with Gaussian string tension fluctuations (12). Therefore, the Hagedorn function in Equation (30) can be rewritten as

(33) $${\left(1+\frac{p_{T}}{p_{0}}\right)}^{-m}=\int_{0}^{\infty }\int_{0}^{\infty }\mathrm{SM}\left(p_{T},x\right)\;P_{G}\left(x,T\right)\;T_{\mathrm{Hag}}\left(T\right)\;dx\;dT,$$

where $T_{\mathrm{Hag}}\left(T\right)$ is given by Equation (31).

Notice that the integration of the joint probability $P_{G}\left(x\right)\;T_{\mathrm{Hag}}\left(T\right)$ over *T* yields the string tension fluctuations associated with the Hagedorn function, making it compatible with the string fragmentation picture. Let us discuss the procedure in detail. Note that the Gaussian distribution is directly expressed in terms of *T*, with $T=\varsigma /\sqrt{2\pi }$, so that

(34) $$P_{G}\left(x\right)=\frac{1}{\pi T}\exp\left(-\frac{x^{2}}{4\pi T^{2}}\right).$$

Thus, we have

(35) $$\begin{array}{cc} P_{\mathrm{Hag}}\left(x\right)\; & =\int_{0}^{\infty }P_{G}\left(x\right)\;T_{\mathrm{Hag}}\left(T\right)\;dT\\ \; & =\frac{p_{0}^{m}}{\pi \Gamma (m)}\int_{0}^{\infty }e^{-p_{0}/T}e^{-x^{2}/4\pi T^{2}}T^{-(m+2)}\;dT, \end{array}$$

Introducing s=1/T, we can rewrite Equation (35) as

(36) $$\begin{array}{cc} P_{\mathrm{Hag}}\left(x\right)\; & =\frac{p_{0}^{m}}{\pi \Gamma (m)}\int_{0}^{\infty }s^{m}\exp\left[-p_{0}s-\lambda s^{2}\right]ds,\;\mathrm{with}\;\lambda \equiv \frac{x^{2}}{4\pi }, \end{array}$$

which is a standard parabolic integral, defined as [40]

(37) $$\int_{0}^{\infty }s^{\nu }\;\exp\left(-\beta s-\lambda s^{2}\right)\;ds=\Gamma \left(\nu +1\right)\;{\left(2\lambda \right)}^{-(\nu +1)/2}\;\exp\left(\beta^{2}/8\lambda \right)\;D_{-(\nu +1)}\;\left(\frac{\beta }{\sqrt{2\lambda }}\right),$$

where $D_{-\nu }\left(z\right)$ is the parabolic cylinder function of order −ν defined as

(38) $$D_{-\nu }\left(z\right)=\frac{\exp(-z^{2}/4)}{\Gamma (\nu )}\int_{0}^{\infty }\exp\left(-zt\right)t^{\nu -1}\exp\left(-t^{2}/2\right)dt,$$

with ν>0 and z>0. Thus, the string tension fluctuations associated with the Hagedorn function are

(39) $$P_{\mathrm{Hag}}\left(x\right)=\frac{m\;p_{0}^{m}}{\pi }\;{\left(2\lambda \right)}^{-(m+1)/2}\;\exp\left(p_{0}^{2}/8\lambda \right)\;D_{-(m+1)}\left(\frac{p_{0}}{\sqrt{2\lambda }}\right).$$

Interestingly, the parabolic cylinder $D_{-\nu }\left(z\right)$ function is related to the Tricomi function (22) through

(40) $$U\left(a,\frac{1}{2},z\right)=z^{\frac{1}{4}-\frac{a}{2}}\;\exp\left(z/2\right)\;D_{-2a}\left(\sqrt{2z}\right).$$

Therefore, the Hagedorn function can equivalently be written as [36]

(41) $${\left(1+\frac{p_{T}}{p_{0}}\right)}^{-m}=\int_{0}^{\infty }\mathrm{SM}\left(p_{T},x\right)\;P_{\mathrm{Hag}}\left(x\right)\;dx.$$

with

(42) $$P_{\mathrm{Hag}}\left(x\right)=\frac{m\;\pi^{\frac{m-1}{2}}\;p_{0}^{m}}{x^{m+1}}\;U\;\left(\frac{m+1}{2},\frac{1}{2},\frac{\pi p_{0}^{2}}{x^{2}}\right),$$

implying that the Hagedorn function also arises from the picture of color string fragmentation.

Let us examine the asymptotic behaviors of $P_{\mathrm{Hag}}\left(x\right)$. Using the asymptotic limits of the U function in Equation (24) with a=(m+1)/2 and $z=\pi p_{0}^{2}/x^{2}$ and considering the factor $x^{-(m+1)}$, we find that at x→0, $P_{\mathrm{Hag}}\left(x\right)$ takes the form

(43) $$P_{\mathrm{Hag}}\left(x\right)=\frac{m\;\pi^{(m-1)/2}p_{0}^{m}}{\Gamma (1+m/2)}\left[1-\frac{\left(m+1\right)\left(m+2\right)x^{2}}{4\pi p_{0}^{2}}+O\left(x^{4}\right)\right],$$

implying that $P_{\mathrm{Hag}}\left(x\right)$ behaves as a Gaussian distribution with variance $\varsigma_{\mathrm{Hag}}^{2}=2\pi p_{0}^{2}/\left[\left(m+1\right)\left(m+2\right)\right]$ at small string tension values [36]. On the other hand, for x→∞, we find

(44) $$P_{\mathrm{Hag}}\left(x\right)\propto x^{-(m+1)}\left(1-\sqrt{4\pi p_{0}^{2}}\frac{\Gamma \left(\frac{m}{2}+1\right)}{\Gamma \left(\frac{m+1}{2}\right)}x^{-1}+O\left(x^{-2}\right)\right).$$

Note that the leading contribution comes from the factor $x^{-(m+1)}$, exhibiting a power-law tail at large string tension values, confirming it is a heavy-tailed distribution.

The methodology discussed for the Hagedorn function can be used for determining the string tension fluctuations associated with a model with known temperature fluctuations. For models with a known distribution describing string tension fluctuations, the inverse pathway can be used to obtain the temperature fluctuations. In Section 4.3, we discuss this procedure for the Tricomi function.

### 4.3. Temperature Fluctuations of the Tricomi Function

Similar to the case discussed in Section 4.2, it is possible to derive a temperature fluctuation description for the *U* function. To this end, we seek a function T(T) such that their convolution with the Gaussian string tension fluctuations yields the *q*-Gaussian distribution, that is,

(45) $$P_{qG}\left(x\right)=\int_{0}^{\infty }P_{G}\left(x\right)\;T_{U}\left(T\right)\;dT.$$

Note that

(46) $${\left(1+\left(q-1\right)\frac{x^{2}}{2\sigma^{2}}\right)}^{\frac{1}{1-q}}=\int_{0}^{\infty }\Gamma \;\left(s;\frac{1}{q-1},\frac{2\sigma^{2}}{q-1}\right)\;\exp\left(-x^{2}s\right)\;ds.$$

By introducing the change of variables $s=1/(4\pi T^{2})$, we find

(47) $$\begin{array}{cc} {\left(1+\left(q-1\right)\frac{x^{2}}{2\sigma^{2}}\right)}^{\frac{1}{1-q}}\; & =\int_{0}^{\infty }\Gamma \;\left(\frac{1}{4\pi T^{2}};\frac{1}{q-1},\frac{2\sigma^{2}}{q-1}\right)\;\exp\left(-\frac{x^{2}}{4\pi T^{2}}\right)\;\frac{dT}{2\pi T^{3}}. \end{array}$$

Thus, we identify the temperature fluctuations of the Tricomi function as (see Appendix B for details)

(48) $$T_{U}\left(T\right)=\frac{2}{T^{3}}\;\Gamma \left(\frac{1}{T^{2}};\frac{1}{q-1}-\frac{1}{2},\frac{\sigma^{2}}{2\pi (q-1)}\right).$$

Hence, the Tricomi spectrum (23) can be rewritten in the form

(49) $$\begin{array}{cc} W_{U}\left(p_{T}\right)\; & =\int_{0}^{\infty }\exp\left(-p_{T}/T\right)\;T_{U}\left(T\right)\;dT. \end{array}$$

**Figure 2:**
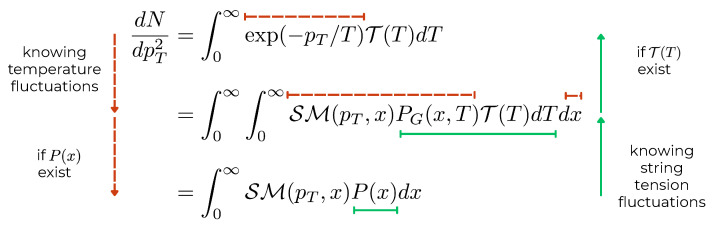
Schematic representation of the complementary pathways connecting string tension fluctuations and temperature fluctuations. For the dashed path, we start from the known temperature fluctuation distribution T(T) and construct the corresponding string tension fluctuations P(x) (if it is integrable) through the thermal spectrum $\exp\left(-p_{T}/T\right)=\int_{0}^{\infty }\mathrm{SM}\left(p_{T},x\right)P_{G}\left(x\right)\;dx$. For the solid path, we follow the reverse direction: starting from the heavy-tailed string tension fluctuations P(x), we derive the temperature fluctuation distribution T(T) (if it exists) by deconvoluting the Gaussian kernel from the thermal spectrum. Brackets indicate which distributions are known (starting points) versus derived (endpoints) for each pathway.

In consequence, both Hagedorn and Tricomi approaches can be expressed as convolutions of the thermal distribution with temperature fluctuations. Interestingly, both Equations (30) and (49) are Laplace-type transforms of $T_{\mathrm{Hag}}\left(T\right)$ and $T_{U}\left(T\right)$, respectively. In Figure 2, we illustrate the methodology used for both the Hagedorn and Tricomi functions for determining their corresponding string tension and temperature fluctuations, which can be applied to study other models.

In both approaches, heavy-tailed string tension fluctuations imply that the system departs from the thermal description (15), accounting for the production of high-$p_{T}$ particles as rare events whose probability increases as *q* increases (or *m* decreases). Additionally, $T_{\mathrm{Hag}}\left(T\right)$ and $T_{U}\left(T\right)$ can be interpreted as local temperature fluctuations occurring in small regions. In particular, high-$p_{T}$-particle production is associated with very intense color interactions given by high string tension values, such as in the case of hard-gluon emission. In this context, these rare events corresponding to the production of heavy and high-$p_{T}$ particles are associated with values of the random variable *T* in the tail of the temperature fluctuations (as we will discuss in Section 4.7), which may be identified as sites with very high local temperatures [36].

We must emphasize that the nonextensive character of the initial state leads to a system of produced particles that is out of thermal equilibrium. However, both $P_{U}$ and $P_{\mathrm{Hag}}$ behave as Gaussian distributions at low string tension values, allowing us to properly define the temperature for the Hagedorn and Tricomi approaches. This characteristic becomes a mandatory requirement for the existence of the temperature fluctuations of any model attempting to describe the $p_{T}$ spectrum.

**Figure 3:**
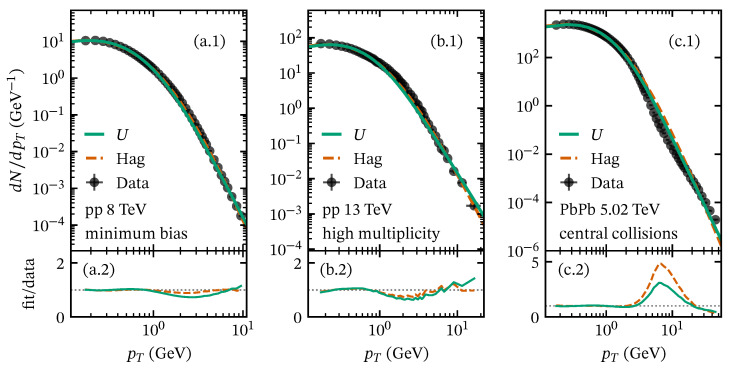
Samples of fits to the transverse momentum spectrum of charged particles in pp and PbPb collisions (ALICE data [34,41,42]) using the Tricomi function (solid lines) and the Hagedorn function (dashed lines). (**a.1**) Minimum-bias pp at $\sqrt{s}=13$ TeV. (**b.1**) High-multiplicity pp at $\sqrt{s}=13$ TeV (SPD I’ class). (**c.1**) Central (0–5%) PbPb collisions at $\sqrt{s_{\mathrm{NN}}}=5.02$ TeV. The lower panels (**a.2**–**c.2**) show their corresponding fit-to-data ratios.

To assess the validity of the *U* and the Hagedorn/Tsallis functions, we analyze some datasets of the $p_{T}$ spectrum of charged particles produced in pp and PbPb reported by the ALICE Collaboration [34,41,42] using Equations (23) and (28). Samples of the fits to data are shown in Figure 3. For minimum-bias pp collisions, both approaches accurately describe the $p_{T}$ spectrum across the full reported $p_{T}$ range, as shown in Figure 3a.1,a.2 for $\sqrt{s}=8$ TeV. However, in high-multiplicity pp events [41], we observe significant deviations at intermediate $p_{T}$ ($1<p_{T}<6$ GeV), as shown in the fit-to-data ratio in Figure 3b.2 for the SPD I’ class. The deviations are more pronounced in central heavy-ion collisions, as we show in Figure 3c.1,c.2 for the most central PbPb collisions (0–5%) at $\sqrt{s_{NN}}=$ 5.02 TeV [42]. The origin of this deviation may stem from collective phenomena that redistribute transverse momentum, mechanisms that the fragmentation of color strings cannot describe. The main effect of collective phenomena in the $p_{T}$ spectrum is the enhancement in particle production in the intermediate region as a consequence of particles interacting in the presence of a medium. Examples of such phenomena include jet quenching, color reconnection, energy loss, and radial flow, among others. Considering all the above, it is necessary to develop models that explain particle production beyond the color string fragmentation mechanism. One way to incorporate these effects into the description of charged-particle production is to combine the Tallis approach with additional assumptions. For instance, using the blast-wave model provides insights into collective effects and relates them to a nonextensivity property of the system [43,44,45,46]. Other perspectives include modeling the collective phenomena as an independent contribution to the $p_{T}$ spectrum considering the string fragmentation as a baseline for particle production [23], as we further discuss in Section 4.4.

### 4.4. Softened-Hadron Production Beyond String Fragmentation

In the previous section, we found that the Schwinger mechanism with heavy-tailed string tension fluctuations gives rise to a function that describes the entire $p_{T}$ spectrum of the particles produced in ultrarelativistic collisions. However, the color string fragmentation does not account for other effects that modify the $p_{T}$ spectrum, for instance, well-known collective phenomena such as radial flow, jet quenching, color reconnection, energy loss, and other effects that redistribute the $p_{T}$ of the produced particles.

Recently, in Ref. [23], the authors conducted a data-driven study of the hadron production that arises from mechanisms beyond string fragmentation. The analysis of the $p_{T}$ spectrum data from pp to AA collisions at LHC energies reveals that the differences between the Tricomi function and the experimental data can be collapsed into a $p_{T}$-exponential trend independently of center-of-mass energy, multiplicity, centrality classification, or system size. Consequently, the complete $p_{T}$ spectrum is proposed to be composed of a color string fragmentation baseline (given by the *U* function) and an exponential term. Thus

(50) $$\frac{dN}{dp_{T}^{2}}=A_{U}U\left(\frac{1}{q_{S}-1}-\frac{1}{2},\frac{1}{2},\frac{\pi p_{T}^{2}\left(q_{S}-1\right)}{2\sigma_{S}^{2}}\right)+A_{\mathrm{th}}\exp\left(-p_{T}/T_{\mathrm{th}}\right),$$

where $T_{\mathrm{th}}$ corresponds to the $p_{T}$ scale related to the softened-hadron production. We use the subscript *S* to differentiate the parameters in Equation (50) from those in Equation (23). In Equation (50), $A_{U}$ is the weight of the produced particles directly coming from the string fragmentation process. The second term accounts for the production of particles that redistribute their $p_{T}$ due to collective phenomena [23].

Note that Equation (50) exhibits exponential behavior at low $p_{T}$ with effective temperature:

(51) $$T_{S}^{-1}=\frac{1}{A_{U}^{*}+A_{\mathrm{th}}}\left(\frac{A_{U}^{*}}{T_{U}}+\frac{A_{\mathrm{th}}}{T_{\mathrm{th}}}\right),$$

where $A_{U}^{*}=\sqrt{\pi }\;A_{U}/\Gamma \left(a_{q}+1/2\right)$, with $a_{q}=1/\left(q_{S}-1\right)-1/2$. Here $T_{U}$ must be evaluated in $q_{S}$ and $\sigma_{S}$. The effective temperature $T_{S}$ represents the soft scale of the complete spectrum, accounting for both string fragmentation and collective contributions [23]. At high $p_{T}$, Equation (50) behaves according to ${\left(p_{T}^{2}\right)}^{\frac{1}{1-q_{S}}+\frac{1}{2}}$, indicating that only the string fragmentation term contributes to high-$p_{T}$-particle production.

The string tension fluctuations associated with Equation (50) are obtained by adding a *q*-Gaussian and a Gaussian string tension fluctuation

(52) $$P_{S}\left(x\right)=\frac{A_{U}^{*}}{A_{U}^{*}+A_{\mathrm{th}}}\;P_{qG}\left(x;q_{S},\sigma_{S}\right)+\frac{A_{\mathrm{th}}}{A_{U}^{*}+A_{\mathrm{th}}}\;P_{G}\left(x;T_{\mathrm{th}}\right),$$

which is a weighted sum of the distributions defined in Equations (17) and (12).

The asymptotic behaviors of Equation (52) follow from the weighted contribution of each term. At low string tension values, we obtain

(53) $$\begin{array}{ccc} P_{S}\left(x\right) & \propto & \frac{A_{\mathrm{th}}}{\pi T}\left(1-\frac{x^{2}}{4\pi T^{2}}+O\left(x^{4}\right)\right)+\frac{A_{U}\sqrt{2(q_{S}-1)}}{\sigma \Gamma (a_{q_{S}})}\left(1-\frac{x^{2}}{2\sigma_{S}^{2}}+O\left(x^{4}\right)\right) \end{array}$$

(54) $$\begin{array}{ccc} & \propto & 1-\frac{x^{2}}{2\beta_{S}^{2}}+O\left(x^{4}\right), \end{array}$$

resembling the asymptotic behavior of a Gaussian distribution with variance $\beta_{S}^{2}$ given by

(55) $$\beta_{S}^{2}=\left(\frac{A_{\mathrm{th}}}{\pi T}+\frac{A_{U}\sqrt{2(q_{S}-1)}}{\sigma \Gamma (a_{q_{S}})}\right)\frac{2\pi^{2}\sigma_{S}^{2}T^{3}\Gamma \left(a_{q_{S}}\right)}{A_{\mathrm{th}}\sigma_{S}^{3}\Gamma \left(a_{q_{S}}\right)+2\pi^{2}T^{3}A_{U}\sqrt{2(q_{S}-1)}},$$

which combines the contributions from both Gaussian and *q*-Gaussian components.

At high string tension values, the *q*-Gaussian tail dominates:

(56) $$P_{S}\left(x\right)\propto x^{\frac{2}{1-q_{S}}}.$$

The power-law tail in the string tension fluctuations reinforces the discussion of the departure from equilibrium of the system created in ultrarelativistic collisions, signaling the nonextensive behavior encoded in the initial state [23].

Additionally, the temperature fluctuations are

(57) $$T_{S}\left(T\right)=\frac{A_{U}^{*}}{A_{U}^{*}+A_{\mathrm{th}}}\;T_{U}\left(T;q_{S},\sigma_{S}\right)+\frac{A_{\mathrm{th}}}{A_{U}^{*}+A_{\mathrm{th}}}\;\delta \left(T-T_{\mathrm{th}}\right),$$

where $T_{U}$ is given by Equation (48) and the Dirac delta $\delta (T-T_{\mathrm{th}})$ arises from the thermal-like component.

The delta function indicates that a fraction of observed particles redistributes their $p_{T}$ as a $p_{T}$ exponential with a characteristic scale $T_{\mathrm{th}}$. This could be interpreted as a bunch of “thermalized” particles by collective phenomena such as radial flow, long-range angular correlations, and the suppression of high-$p_{T}$ particles that are characteristics of QGP formation [47,48,49,50,51,52,53,54,55,56]. However, the presence of the Gamma-distributed component $T_{U}\left(T\right)$ indicates that the global system remains out of thermal equilibrium, with significant temperature variations along the system.

### 4.5. String Tension Fluctuations from a Percolation Picture

It is noteworthy that generalized Gamma distributions are widely used in contexts with conformal symmetry. For instance, the Color String Percolation Model (CSPM) provides geometric arguments for constructing string tension fluctuations based on the clustering of color sources [7]. In this framework, as centrality increases (or equivalently, as string density grows), isolated strings merge into larger clusters through percolation. The cluster size distribution is described by a Gamma function, which emerges as a stable fixed point under renormalization group transformations of the form $P\left(x\right)\to x^{n}P\left(x\right)/\langle x^{n}\rangle$ (analogous to Wilson-type block transformations) [7,57,58].

This invariance physically corresponds to the fractal structures in the clustering of strings in the percolation threshold, meaning that the cluster size distribution remains Gamma-distributed because of the self-similarity of clusters in percolation theory [7]. The Gamma distribution of the string tension fluctuation $P_{\Gamma }\left(x\right)$ resulting from the analysis of the cluster sizes is given by [7]

(58) $$P_{\Gamma }\left(x\right)=\frac{2}{x^{3}}\;\Gamma \left(\frac{1}{x^{2}};k,\pi kT_{h}^{2}\right),$$

where *k* and $T_{h}$ are parameters related to hard-particle production. Note that Equation (58), at high string tension values, behaves according to $P_{\Gamma }\left(x\right)\propto x^{-2k-1}$, i.e., it is a heavy-tailed distribution.

Now, the $p_{T}$ spectrum is

(59) $$\begin{array}{ccc} \frac{dN}{dp_{T}^{2}} & \propto & 2\int_{0}^{\infty }\Gamma \left(\frac{1}{x^{2}};k,\pi kT_{h}^{2}\right)\;\exp\left(-\pi p_{T}^{2}/x^{2}\right)\;\frac{dx}{x^{3}}. \end{array}$$

Using the result of Appendix A and the change of variable $s=\pi /x^{2}$, we obtain

(60) $$\frac{dN}{dp_{T}^{2}}\propto \int_{0}^{\infty }\Gamma \left(s;k,kT_{h}^{2}\right)\;\exp\left(-p_{T}^{2}s\right)\;ds={\left(1+\frac{p_{T}^{2}}{kT_{h}^{2}}\right)}^{-k},$$

which resembles a Hagedorn function with argument $p_{T}^{2}$ [59,60].

Equation (60) exhibits power-law behavior $\propto {\left(p_{T}^{2}\right)}^{-k}$ at high $p_{T}$. However, at low $p_{T}$, the first term contributing to the series expansion yields

(61) $${\left(1+\frac{p_{T}^{2}}{kT_{h}^{2}}\right)}^{-k}\propto 1-\frac{p_{T}^{2}}{T_{h}^{2}}+O\left(p_{T}^{4}\right),$$

which has a Gaussian form in $p_{T}$ rather than the required thermal distribution.

While the CSPM offers a QCD-inspired interpretation based on the geometric clustering of color sources, our phenomenological approach focuses on extracting effective fluctuation distributions directly from the $p_{T}$ spectrum. For models with well-defined soft scales (e.g., Tricomi and Hagedorn), this approach successfully relates string tension and temperature fluctuations. However, not all phenomenological models admit valid descriptions of temperature fluctuations.

### 4.6. Fluctuations of the Two-Component Model

The two-component model was proposed for describing the soft and hard parts of the $p_{T}$ spectrum by considering an exponential decay plus a quasi-power-law function modeling soft and hard contributions, respectively, which reads [22,61,62,63]

(62) $$\frac{dN}{dp_{T}^{2}}\propto W_{2c}=A_{s}\exp\left(-p_{T}/T_{s}\right)+A_{h}{\left(1+\frac{p_{T}^{2}}{kT_{h}^{2}}\right)}^{-k},$$

where $A_{s}$, $T_{s}$, $A_{h}$, *k*, and $T_{h}$ are the fitting parameters. The two contributions in Equation (62) assure the compatible definition of the effective temperature for our framework and inherit the properties of the Gamma distribution for the string tension fluctuations.

Now, we can recover the thermal behavior of $W_{2c}$ at low $p_{T}$ as

(63) $$\begin{array}{cc} W_{2c}\; & \approx A_{s}+A_{h}-\frac{A_{s}}{T_{s}}\;p_{T}+O\left(p_{T}^{2}\right)\\ & \approx \left(A_{s}+A_{h}\right)\;\exp\;\left(-\frac{p_{T}}{T_{2c}}\right), \end{array}$$

with an effective soft scale

(64) $$T_{2c}=\left(1+\frac{A_{h}}{A_{s}}\right)T_{s}.$$

Notice that the hard part of Equation (62) does not contribute to the linear term in the low-$p_{T}$ expansion, only the constant term. On the other hand, Equation (62) inherits the power law from the hard component $\propto {\left(p_{T}^{2}\right)}^{-k}$ at high $p_{T}$ values. Therefore, the two-component model is a heavy-tailed distribution with a soft scale defined mainly through the exponential term in Equation (62).

Following our results discussed above, we can write the string tension fluctuations associated with the two-component model as

(65) $$P_{2c}\left(x\right)=\frac{A_{s}}{\left(A_{s}+A_{h}\right)\pi T_{s}}\exp\left(-\frac{x^{2}}{4\pi T_{s}^{2}}\right)+\frac{2A_{h}}{\left(A_{s}+A_{h}\right)x^{3}}\Gamma \left(\frac{1}{x^{2}};k,\pi kT_{h}^{2}\right).$$

Interestingly, $P_{2c}\left(x\right)$ has independent asymptotic behaviors that correspond to the Gaussian and the Gamma terms in Equation (65), which we discussed in previous sections, giving

(66) $$P_{2c}\left(x\right)\propto \left\{\begin{array}{cc} 1-\frac{x^{2}}{4\pi T_{s}^{2}}+O\left(x^{4}\right),\; & x\to 0,\\ x^{-2k-1},\; & x\to \infty , \end{array}\right.$$

meaning that the distribution describing the string tension fluctuations of the two-component model is heavy-tailed.

On the other hand, in the computation of the temperature fluctuations, the $p_{T}$-exponential term contributes with a Dirac’s delta function. In contrast, the hard component can be constructed by applying the inverse Laplace transform to map string tension fluctuations back to the corresponding temperature distribution, using an appropriate change of variables. This procedure yields

(67) $$T_{2c}\left(T\right)=\frac{2^{\frac{1}{2}-k}\;\sqrt{\pi }}{\Gamma (k)}\;k^{\frac{k}{2}+\frac{1}{4}}\;T_{h}^{\;k+\frac{1}{2}}\;T^{-k-\frac{3}{2}}\;J_{k-\frac{1}{2}}\;\left(\frac{\sqrt{k}\;T_{h}}{T}\right),$$

where $J_{k-1/2}$ is a Bessel function of the first kind. By construction, integrating $T_{2c}\left(T\right)$ with Equation (34) in *T* recovers the Gamma distribution in $1/x^{2}$.

Although $T_{2c}\left(T\right)$ is a solution, this function is not a probability density because Bessel functions oscillate around zero with a nonconvergent integral. Thus, the Bessel function in Equation (67) changes its sign as *T* decreases, implying that $T_{2c}\left(T\right)$ also changes sign. Thus, $T_{2c}\left(T\right)$ cannot be taken as a probability distribution describing temperature fluctuations.

Our derivation reveals that the found representation involves oscillatory weights given by Equation (67). This limitation arises from the hard component in Equation (62) that depends on $p_{T}^{2}$ rather than $p_{T}$, which makes it impossible to determine the temperature as the slope of the $p_{T}$ spectrum at low $p_{T}$.

We must point out that this analysis establishes a requirement for the validity of temperature fluctuation descriptions: a model that admits a well-defined temperature fluctuation distribution T(T) as a valid probability density needs to exhibit exponential behavior $\exp(-p_{T}/T_{\mathrm{eff}})$ at low $p_{T}$. For instance, the $m_{T}$-exponential distribution cannot be expressed as the convolution of the thermal distribution with temperature fluctuations, since their low-$p_{T}$ expansion depends on both *m* and $p_{T}$ in a way that prevents factorization. Similarly, although Equation (60) can be derived from string tension fluctuations, it does not recover the thermal description at low $p_{T}$ due to its quadratic dependence on $p_{T}$; consequently, neither this function nor the two-component model admits valid temperature fluctuation descriptions within our framework. This asymmetry demonstrates that while string tension fluctuations can exist for the models studied here, the derivation of the temperature fluctuations is more restrictive, limited to models with proper thermal low-$p_{T}$ behavior.

### 4.7. Pareto-Type Distribution

Let us close this section by studying the general origin of power-law behavior in the $p_{T}$ distribution. We have discussed that heavy-tailed string tension fluctuations play a crucial role in determining the shape of the $p_{T}$ spectrum via the Schwinger mechanism. Consider the case where these fluctuations follow a Pareto-type distribution $P_{p}\left(x\right)\propto x^{-(\alpha +1)}$. When such fluctuations are convoluted with the Schwinger mechanism (using the definition of the Gamma function), the resulting $p_{T}$ spectrum exhibits a power-law tail:

(68) $$\int_{0}^{\infty }\mathrm{SM}\left(p_{T},x\right)\frac{dx}{x^{\alpha +1}}\propto p_{T}^{-\alpha }.$$

This result generalizes the asymptotic behavior at high $p_{T}$ values observed in all models discussed above. Just as the effective temperature $T_{\mathrm{eff}}$ characterizes the soft scale (low-$p_{T}$-exponential behavior), the exponent α governs the high-$p_{T}$ power-law tail through the Pareto-type behavior $∼x^{-(\alpha +1)}$. Both the power-law exponent and the effective temperature parameters are summarized in Table 1 for all models discussed in this work.

A main implication of relation (68) is that hard-particle production arises from rare but significant fluctuations toward high string tensions, corresponding to hard-partonic processes such as high-intensity color-field configurations or energetic gluon emissions. The Pareto distribution $P_{p}\left(x;\alpha \right)=\alpha x_{m}^{\alpha }/x^{\alpha +1}$ has a natural lower cutoff $x>x_{m}$, suggesting a characteristic string tension scale separating soft and hard production mechanisms. This scale depends on the hard process cutoff, which must be determined by deep analysis. In this sense, the hard scale discussed in modern phenomenological applications [9,22,61,62,63] plays a role analogous to a transition momentum between soft-particle production and hard-scattering processes. However, our phenomenological methodology cannot fully disentangle the underlying processes that are involved in the $p_{T}$ distribution, particularly at intermediate $p_{T}$, where collective phenomena become important [23]. A complete description would require integrating hard-QCD matrix elements, collective flow effects, and string fragmentation—a challenge that remains open.

Note that the power law $p_{T}$ spectrum can also be obtained as

(69) $$\int_{0}^{\infty }\exp\left(-p_{T}/T\right)\frac{dT}{T^{\alpha +1}}\propto p_{T}^{-\alpha },$$

meaning that the temperature fluctuations of the Pareto-type string tension fluctuations are given by $T\propto T^{-\alpha -1}$, which is also a power law. However, as discussed above, the cutoff in the Pareto distribution indicates that the thermal behavior in the $p_{T}$ spectrum $\propto p_{T}^{-\alpha }$ and the Gaussian behavior in the string tension fluctuations $P_{p}\left(x\right)$ cannot be recovered, which is the requirement for having well-defined temperature fluctuations.

Note that similar to the Gamma distribution, the Pareto distribution also remains invariant under transformations of the type xP(x)/〈x〉, specifically

(70) $$\frac{xP_{p}\left(x;\alpha \right)}{\langle x\rangle }=\frac{x}{\langle x\rangle }\cdot \frac{\alpha x_{m}^{\alpha }}{x^{\alpha +1}}=\frac{\left(\alpha -1\right)x_{m}^{\alpha -1}}{x^{\alpha }}=P_{p}\left(x;\alpha -1\right),$$

but it also it satisfies $x^{n}P_{p}\left(x;\alpha \right)/\langle x^{n}\rangle =P_{p}\left(x;\alpha -n\right)$ for n<α. This scale invariance of the Pareto distribution demonstrates that the heavy-tailed string tension fluctuation distributions analyzed in this work satisfy Koba–Nielsen–Olesen (KNO) scaling in the high-$p_{T}$ regime [64,65], which reflects the self-similar structure of hard-particle production modulated by the exponent α across different hard processes.

## 5. Data Analysis and Discussion

We apply the framework developed in Section 3 and Section 4 to analyze the experimental data of charged-particle production at LHC energies. The datasets studied correspond to experimental data on the midrapidity $p_{T}$ spectrum for minimum-bias pp and pPb collisions at different center-of-mass energies reported by the ALICE Collaboration in Ref. [34]. We also consider data under multiplicity classification for pp collisions at 13 TeV [41] and centrality classifications for PbPb collisions at 5.02 TeV [42].

The fitting procedure differs for each model depending on the physical region it describes. For the thermal distribution, which captures the low-$p_{T}$ regime dominated by soft-particle production, we perform a scan to determine the cutoff $p_{T}^{\max}$ that minimizes $\chi^{2}$/NDF, determining the range where the thermal description is valid. In Figure 1, we show the performance of the thermal distribution in describing the low-$p_{T}$ region of the $p_{T}$ spectrum for minimum-bias pp collisions. The obtained parameters of the thermal distribution are reported in Table A1.

For the Tricomi and Hagedorn functions, which are designed to describe the full $p_{T}$ spectrum including the power-law tail at high $p_{T}$, the fitting procedure accounts for isolating the contributions from soft and hard processes. To this end, we excluded an intermediate-$p_{T}$ interval during the fit, typically in the range $p_{T}∼1$-6 GeV, and sought the region that minimizes $\chi^{2}$/NDF. This procedure ensures that the fitted functions accurately capture both the low- and high-$p_{T}$ regimes, avoiding contamination from particles produced by collective phenomena. The corresponding fitting parameters of the Tricomi and Hagedorn functions are reported in Table A2 and Table A3, respectively. In Figure 3, we show representative cases of these fits using the Hagedorn and Tricomi functions, exhibiting good performance for small systems, as evidenced by the fit-to-data ratios in Figure 3a.2,b.2. However, for heavy-ion collisions, the Hagedorn and Tricomi functions deviate from experimental data in the intermediate-$p_{T}$ region. In Figure 3c.2, we show these deviations in the fit-to-data ratio, revealing that the Hagedorn function overestimates the experimental data by up to a factor of five in the most central (0–5%) PbPb collisions at 5.02 TeV.

**Figure 4:**
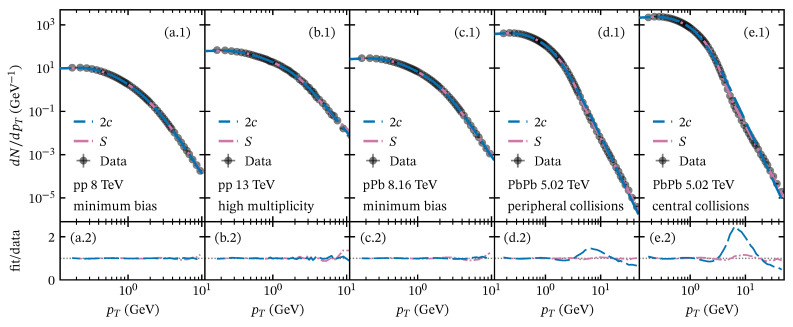
Transverse momentum spectrum of charged particles at midrapidity for various collision systems: (**a.1**,**a.2**) minimum-bias pp collisions at $\sqrt{s}=8$ TeV; (**b.1**,**b.2**) high-multiplicity events in pp collisions at $\sqrt{s}=13$ TeV (SPD I’ class); (**c.1**,**c.2**) minimum-bias pPb at $\sqrt{s_{\mathrm{NN}}}=8.16$ TeV; (**d.1**,**d.2**,**e.1**,**e.2**) PbPb collisions at $\sqrt{s_{\mathrm{NN}}}=5.02$ TeV for centrality classes 40–50% and 0–5%, respectively. The upper panels show fits to the $p_{T}$ spectrum using the two-component model (dashed lines) and the description beyond string fragmentation (dash-dotted lines). Lower panels display the corresponding fit-to-data ratios.

We use Equation (50) to study the contribution of collective phenomena to the $p_{T}$ spectrum, which accounts for these effects [23]. In Figure 4, we show sample fits of Equation (50) and the two-component model (62) to full experimental $p_{T}$ spectrum data across different collision systems. The fitting parameters for both models are reported in Table A4 and Table A5, respectively.

Note that the model incorporating the contribution of fragmentation and collective phenomena mechanisms (Equation (50)) provides an excellent performance in describing the $p_{T}$ spectrum across the full $p_{T}$ range. The weights determined for the components of the softened-hadron production reflect a non-negligible contribution to the transverse momentum spectrum in the intermediate-$p_{T}$ region, which increases with multiplicity and centrality [23]. We emphasize that the processes that produce softened hadrons differ from the soft and hard components already incorporated into the *U* function.

In the two-component model, the contributions of the soft and hard parts of the $p_{T}$ spectrum are considered independent; therefore, it models the two scales without explicitly addressing collective effects. Since the majority of charged-particle production occurs in the soft region, the weight of the soft component is much larger than that of the hard component (see Table A5).

**Figure 5:**
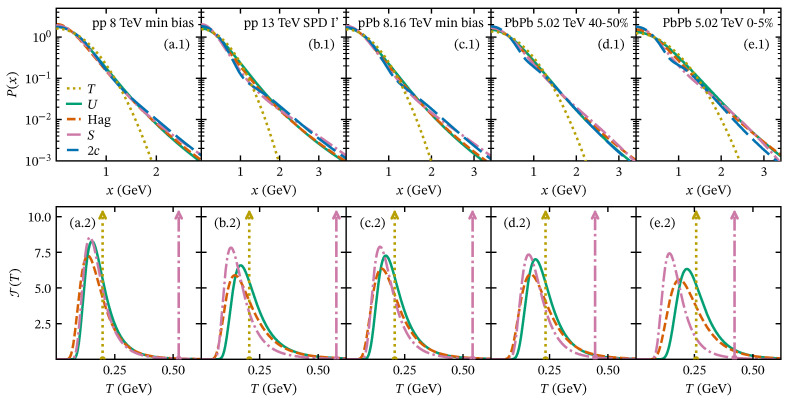
String tension fluctuations P(x) (upper panels) and temperature fluctuations T(T) (lower panels) extracted from fits to charged-particle spectrum in: (**a.1**,**a.2**) minimum-bias pp at $\sqrt{s}=8$ TeV, (**b.1**,**b.2**) high-multiplicity pp at $\sqrt{s}=13$ TeV (SPD I’ class), (**c.1**,**c.2**) minimum-bias pPb at $\sqrt{s_{\mathrm{NN}}}=8.16$ TeV, (**d.1**,**d.2**) peripheral (40–50%) PbPb at $\sqrt{s_{\mathrm{NN}}}=5.02$ TeV, and (**e.1**,**e.2**) central (0–5%) PbPb at $\sqrt{s_{\mathrm{NN}}}=5.02$ TeV. Arrows indicate the Dirac delta function.

In Figure 5, we present our results for the string tension (upper panels) and temperature fluctuations (lower panels) for representative collision systems. The Gaussian distribution (dotted lines) becomes broader as multiplicity, system size, or centrality increases, which is directly linked to the temperature value corresponding to the thermal $p_{T}$ spectrum. As we mentioned previously, this temperature value appears to reflect the weighted sum of hadron masses, taking larger values for heavy-ion collisions due to the increased production of heavier hadrons.

The heavy-tailed string tension fluctuations increase the probability of having strings with higher tension, which is a necessary requirement for the production of high-$p_{T}$ particles. These rare events endow the initial state with a nonextensive description.

In all the models discussed in this manuscript, the increase in the effective temperature corresponds to a greater production of low-$p_{T}$ particles, as expected when center-of-mass energy and multiplicity increase. The production of particles that redistribute their $p_{T}$ arises due to collective effects, which also enhance the value of the effective temperature. This is reflected in the shift in the temperature fluctuation peak toward lower scales and in the broadening of the T(T) distributions from pp to central PbPb collisions, signaling the formation of QGP in heavy-ion collisions.

The model that considers fragmentation and softened-hadron production adequately captures the behavior of the $p_{T}$ spectrum at intermediate $p_{T}$ values more effectively than the two-component model because it provides a continuous description of the transition from non-collective to collective regimes through the soft and hard scales. In contrast, the two-component model introduces a sharp separation between soft and hard regions of the $p_{T}$ spectrum but does not capture the transition to the gradual onset of collectivity signals. This is exhibited in the string tension fluctuations for central PbPb collisions (Figure 5e.1), where the pronounced drop near x∼1 GeV separates the Gaussian and Gamma contributions in the two-component model, whereas the softened model exhibits a smoother transition.

**Figure 6:**
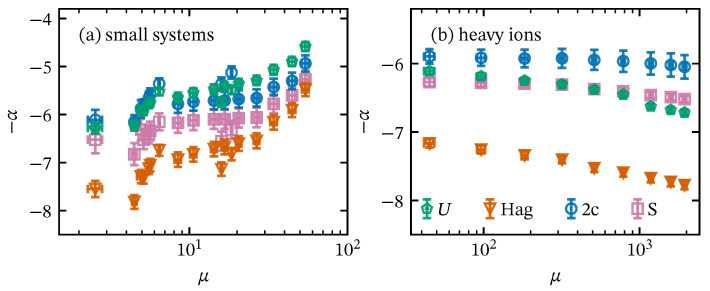
Power-law exponent α for (**a**) small systems and (**b**) heavy ions as a function of average charged-particle multiplicity density μ≡〈dN/dη〉 for the Tricomi function (*U*), Hagedorn function (Hag), two-component (2c), and softened-hadron (*S*) models.

Another physical effect that we can analyze within the framework presented in the manuscript is the suppression of high-$p_{T}$ particles in heavy-ion collisions, which is reflected in the decrement in the exponent −α for the most central collisions, as shown in Figure 6b. In such cases, the partons lose energy as they travel across the quark–gluon plasma, leading to a decrement in high-$p_{T}$-particle production [66]. In contrast, pp collisions show a different trend: as multiplicity increases, −α also increases, indicating an enhancement in the $p_{T}$ of the produced particles. Even though Equation (50) reveals signals of collective phenomena in small systems, they lacks jet quenching [67,68], as shown in Figure 6a.

The enhancement in the production of high-$p_{T}$ particles in pp collisions, captured by α, can be associated with an increase in the number of degrees of freedom in QCD, supported by the studies of the Shannon entropy and heat capacity considering the $p_{T}$ spectrum as a probability density function [20,68]. Since the Schwinger mechanism with string tension fluctuation does not incorporate information beyond the strength of the color interaction, we may conjecture that the increase in the number of degrees of freedom corresponds to a major heavy flavor production. This observation is consistent with the theoretical prediction for α=4–5, obtained for hard scattering by considering the number of colors and flavors of active quarks and assuming other fundamental free-of-scale-QCD conditions [18,69].

## 6. Conclusions

In this work, we studied in detail the production of charged particles in ultrarelativistic collisions through the lens of color string fragmentation. Within this framework, the $p_{T}$ spectrum is governed by the Schwinger mechanism, which is obtained by computing the probability of quantum tunneling for two semiclassical relativistic particles interacting via a linear potential. The fundamental stochastic nature of QCD vacuum excitations is incorporated through admitting fluctuations in the intensity of the color interaction, namely, the string tension.

The main finding reported in this manuscript is the mandatory requirement of using heavy-tailed string tension fluctuations to accurately describe the power-law behavior of the $p_{T}$ spectrum observed experimentally at high $p_{T}$. Similar to the Pareto distribution, the heavy-tailed characteristic is required for the KNO scaling in intense color interactions, making these distributions compatible with renormalization group properties and the study of hard processes. Thus, the initial state becomes nonextensive. If the temperature is well-defined, this characteristic results in a final state that is out of thermal equilibrium due to temperature fluctuations in small regions of the system. We must emphasize that the existence of temperature fluctuations requires string tension fluctuations, yielding a $p_{T}$ spectrum with asymptotic behavior at low $p_{T}$ in the form of a thermal distribution. On the contrary, it is necessary to use alternative temperature definitions to establish consistent thermodynamics.

It is possible to extract valuable physical information within this framework by analyzing the experimental data reported by large collaborations. In particular, we analyzed the data reported by the ALICE Collaboration across different collision systems. We found that in all the models discussed here, the temperature increases with the center of mass energy or multiplicity as a result of a greater production of low-$p_{T}$ hadrons, but also due to the emergence of the production of softened hadrons, which redistribute their $p_{T}$ due to collective phenomena. Additionally, a decreasing behavior of the exponent governing the tail of string tension fluctuations can be directly understood as a decrement in the probability of producing high-$p_{T}$ particles, which is consistent with the suppression of high-$p_{T}$ particles (a main signal of QGP formation), as we observed for heavy-ion collisions, but which is absent in pp collisions.

## Figures and Tables

**Table 1:** The effective temperature $T_{\mathrm{eff}}$ characterizes the soft scale governing low-$p_{T}$-exponential behavior $\exp(-p_{T}/T_{\mathrm{eff}})$. The α exponent that governs the high-$p_{T}$ power-law tail $p_{T}^{-\alpha }$ in the $p_{T}$ spectrum arises from Pareto-type string tension fluctuations $P\left(x\right)∼x^{-(\alpha +1)}$.

Model	$T_{\mathrm{eff}}$	α
Thermal (*T*)	*T* [Equation (16)]	—
Tricomi (*U*)	$T_{U}$ [Equation (26)]	$\frac{2}{q-1}-1$
Hagedorn (Hag)	$T_{\mathrm{Hag}}$ [Equation (29)]	*m*
Two-component (2c)	$T_{2c}$ [Equation (64)]	2k
U+ softened-hadron production (*S*)	$T_{S}$ [Equation (51)]	$\frac{2}{q_{S}-1}-1$

## Data Availability

The data that support the findings of this study are available from the corresponding authors upon reasonable request.

## Appendix A. Gamma Distribution Convoluted with an Exponential Decay

A key mathematical result used throughout Section 3 is the convolution of a Gamma distribution with an exponential function. Consider the Gamma distribution in the rate parametrization

(A1) $$\Gamma \left(s;a,b\right)=\frac{b^{a}}{\Gamma (a)}\;s^{a-1}\;\exp\left(-bs\right),$$

and its convolution with an exponential decay exp(−ps) as Wilk and Włodarczyk presented in their work [21]:

(A2) $$\begin{array}{cc} \int_{0}^{\infty }\Gamma \left(s;a,b\right)\;\exp\left(-ps\right)\;ds\; & =\frac{b^{\;a}}{\Gamma (a)}\int_{0}^{\infty }s^{a-1}\exp\left(-\left(b+p\right)s\right)\;ds\\ \; & =\frac{b^{\;a}}{\Gamma (a)}\int_{0}^{\infty }{\left(\frac{t}{b+p}\right)}^{a-1}\exp\left(-t\right)\;\frac{dt}{b+p},\;\mathrm{where}\;t=\left(b+p\right)s,\\ \; & ={\left(\frac{b}{b+p}\right)}^{a}\frac{1}{\Gamma (a)}\int_{0}^{\infty }t^{a-1}\exp\left(-t\right)\;dt\\ \; & ={\left(\frac{b}{b+p}\right)}^{a},\;\mathrm{using}\;\mathrm{the}\;\mathrm{definition}\;\mathrm{of}\;\Gamma \left(a\right),\\ \; & ={\left(1+\frac{p}{b}\right)}^{-a}. \end{array}$$

This identity is a main result for identifying fluctuation distributions that give rise to *q*-exponential functions.

## Appendix B. Derivation of Distributions

The identification of the temperature fluctuations for the Tricomi function requires the identification of the proper Gamma distribution convoluted with a Gaussian kernel in

(A3) $$P_{qG}\left(x\right)=\sqrt{\frac{2(q-1)}{\pi }}\frac{\Gamma \left(\frac{1}{q-1}\right)}{\sigma \Gamma \left(\frac{1}{q-1}-\frac{1}{2}\right)}{\left[1+\frac{\left(q-1\right)x^{2}}{2\sigma^{2}}\right]}^{\frac{1}{1-q}},$$

using the result of Equation (A2) in Appendix A

PqG(x)=2(q−1)πΓ1q−1σΓ1q−1−12∫0∞Γ14πT2;1q−1,2σ2q−1exp−x24πT2dT2πT3   =2(q−1)/πσΓ1q−1−12q−12σ2−1q−1∫0∞exp−σ22π(q−1)T2(4πT2)1q−1−1PG(x)dT2T2(A4)   =2Γ1q−1−12σ22π(q−1)T21q−1−12∫0∞exp−σ22π(q−1)T2PG(x)dTT(A5)   =∫0∞TU(T)PG(x)dT,

with

(A6) $$T_{U}\left(T\right)=\frac{2}{T^{3}}\;\Gamma \left(\frac{1}{T^{2}};\frac{1}{q-1}-\frac{1}{2},\frac{\sigma^{2}}{2\pi (q-1)}\right).$$

## Appendix C. Fitting Parameters

We report the fitting parameters for all models analyzed in this work. The fitting parameters are obtained from fits to ALICE experimental data for charged-particle transverse momentum spectrum [34,41,42]. For the Tricomi and Hagedorn functions, we indicate the $p_{T}$ interval excluded from the fit to minimize systematic deviations arising from collective phenomena beyond string fragmentation. All fits minimize $\chi^{2}$ per number of degrees of freedom (NDF). The average charged-particle multiplicity is denoted by μ≡〈dN/dη〉.

**Table A1:** Fitting parameters of the thermal model.

	$\sqrt{s}$ (TeV)	Class	μ	*T* (GeV)	$p_{T}^{\max}$ (GeV)	$\chi^{2}$/NDF
pp	13	V0M X	2.5(3)	0.20(1)	0.7	0.097/9
	13	V0M IX	4.9(3)	0.20(2)	0.5	0.007/5
	13	V0M VII	8.5(4)	0.20(2)	0.45	0.010/4
	13	V0M VI	10.6(5)	0.20(2)	0.45	0.020/4
	13	V0M IV	14.3(6)	0.20(3)	0.45	0.043/4
	13	V0M III	16.7(7)	0.20(3)	0.45	0.063/4
	13	V0M II	20.5(8)	0.20(3)	0.45	0.084/4
	13	V0M I	26.6(11)	0.21(3)	0.45	0.104/4
	13	SPD IV’	34.1(17)	0.20(3)	0.45	0.167/4
	13	SPD II’	44.6(22)	0.20(3)	0.45	0.206/4
	13	SPD I’	54.1(27)	0.21(3)	0.45	0.265/4
pp	2.76	Min bias	4.5(2)	0.20(1)	0.6	0.181/7
	5.02	Min bias	5.1(1)	0.20(2)	0.5	0.036/5
	7	Min bias	5.5(1)	0.20(2)	0.45	0.011/4
	8	Min bias	5.7(1)	0.20(2)	0.45	0.006/4
	13	Min bias	6.4(1)	0.20(2)	0.45	0.025/4
pPb	5.02	Min bias	15.9(2)	0.21(2)	0.45	0.032/4
	8.16	Min bias	18.5(2)	0.21(2)	0.45	0.039/4
PbPb	5.02	70–80%	45(3)	0.21(3)	0.45	0.011/4
	5.02	60–70%	96(6)	0.22(3)	0.45	0.010/4
	5.02	50–60%	183(8)	0.23(3)	0.45	0.009/4
	5.02	40–50%	318(12)	0.23(3)	0.45	0.009/4
	5.02	30–40%	512(15)	0.24(3)	0.45	0.009/4
	5.02	20–30%	786(20)	0.25(3)	0.45	0.009/4
	5.02	10–20%	1180(31)	0.25(3)	0.45	0.012/4
	5.02	5–10%	1586(46)	0.25(3)	0.5	0.023/5
	5.02	0–5%	1943(54)	0.26(2)	0.55	0.042/6

**Table A2:** Fitting parameters of the Tricomi function.

	$\sqrt{s}$ (TeV)	Class	μ	σ (GeV)	*q*	$p_{T}$ Excluded Interval (GeV)	$\chi^{2}$/NDF
pp	13	V0M X	2.5(3)	0.35(1)	1.275(4)	[1.15,1.25]	3.26/42
	13	V0M IX	4.9(3)	0.37(1)	1.289(5)	[1.05,5.75]	1.00/20
	13	V0M VII	8.5(4)	0.39(1)	1.301(5)	[1.05,5.25]	2.76/21
	13	V0M VI	10.6(5)	0.40(1)	1.306(5)	[1.05,4.75]	4.61/22
	13	V0M IV	14.3(6)	0.41(1)	1.309(5)	[1.05,5.25]	7.51/21
	13	V0M III	16.7(7)	0.42(1)	1.312(5)	[1.05,4.75]	9.42/22
	13	V0M II	20.5(8)	0.43(1)	1.315(5)	[1.05,4.75]	11.52/22
	13	V0M I	26.6(11)	0.44(1)	1.318(5)	[1.05,4.75]	13.83/22
	13	SPD IV’	34.1(17)	0.43(1)	1.330(5)	[1.05,5.25]	13.06/21
	13	SPD II’	44.6(22)	0.43(1)	1.339(6)	[1.05,5.25]	15.53/21
	13	SPD I’	54.1(27)	0.42(1)	1.358(6)	[1.05,5.25]	11.86/21
pp	2.76	Min bias	4.5(2)	0.39(1)	1.276(4)	[1.25,4.25]	6.14/24
	5.02	Min bias	5.1(1)	0.38(1)	1.289(4)	[1.15,5.25]	4.16/21
	7	Min bias	5.5(1)	0.38(1)	1.295(4)	[1.05,5.25]	3.50/20
	8	Min bias	5.7(1)	0.38(1)	1.296(4)	[1.05,5.75]	2.69/19
	13	Min bias	6.4(1)	0.38(1)	1.307(4)	[1.05,5.75]	5.25/19
pPb	5.02	Min bias	15.9(2)	0.42(1)	1.297(4)	[1.05,5.25]	13.91/20
	8.16	Min bias	18.5(2)	0.42(1)	1.308(4)	[1.05,4.75]	16.69/21
PbPb	5.02	70–80%	45(3)	0.42(1)	1.281(2)	[1.05,4.75]	5.38/36
	5.02	60–70%	96(6)	0.44(1)	1.278(2)	[1.05,3.9]	6.76/38
	5.02	50–60%	183(8)	0.46(1)	1.276(2)	[1.05,3.5]	11.32/40
	5.02	40–50%	318(12)	0.48(1)	1.274(2)	[1.05,3.1]	23.20/42
	5.02	30–40%	512(15)	0.49(1)	1.271(2)	[1.05,3.1]	34.10/42
	5.02	20–30%	786(20)	0.51(1)	1.268(2)	[1.15,5.75]	40.91/35
	5.02	10–20%	1180(31)	0.54(1)	1.262(1)	[3.5,5.75]	73.31/51
	5.02	5–10%	1586(46)	0.54(1)	1.261(2)	[3.3,5.75]	78.73/50
	5.02	0–5%	1943(54)	0.55(1)	1.259(2)	[3.3,5.75]	83.23/50

**Table A3:** Fitting parameters of the Hagedorn function.

	$\sqrt{s}$ (TeV)	Class	μ	$p_{0}$ (GeV)	*m*	$p_{T}$ Excluded Interval (GeV)	$\chi^{2}$/NDF
pp	13	V0M X	2.5(3)	0.97(5)	7.55(17)	−	10.41/44
	13	V0M IX	4.9(3)	1.09(4)	7.29(14)	−	3.50/44
	13	V0M VII	8.5(4)	1.09(5)	6.94(15)	[1.05,3.9]	0.97/24
	13	V0M VI	10.6(5)	1.10(5)	6.83(15)	[1.05,3.9]	1.63/24
	13	V0M IV	14.3(6)	1.11(5)	6.72(15)	[1.05,3.9]	3.36/24
	13	V0M III	16.7(7)	1.13(5)	6.68(15)	[1.05,3.9]	4.50/24
	13	V0M II	20.5(8)	1.14(5)	6.60(15)	[1.05,3.9]	6.06/24
	13	V0M I	26.6(11)	1.17(6)	6.55(16)	[1.05,4.25]	7.63/23
	13	SPD IV’	34.1(17)	1.06(6)	6.16(15)	[1.05,3.9]	8.12/24
	13	SPD II’	44.6(22)	1.03(6)	5.91(15)	[1.05,3.9]	10.73/24
	13	SPD I’	54.1(27)	0.93(5)	5.48(14)	[1.05,3.7]	7.26/25
pp	2.76	Min bias	4.5(2)	1.23(4)	7.82(14)	−	8.57/43
	5.02	Min bias	5.1(1)	1.14(4)	7.32(12)	[1.65,1.95]	6.57/39
	7	Min bias	5.5(1)	1.11(3)	7.13(12)	[1.35,3.3]	4.21/29
	8	Min bias	5.7(1)	1.09(4)	7.05(13)	[1.15,3.9]	1.70/24
	13	Min bias	6.4(1)	1.04(3)	6.75(11)	[1.25,3.9]	4.50/25
pPb	5.02	Min bias	15.9(2)	1.23(4)	7.13(14)	[1.05,4.25]	6.23/22
	8.16	Min bias	18.5(2)	1.19(4)	6.82(13)	[1.05,4.25]	7.41/22
PbPb	5.02	70–80%	45(3)	1.20(2)	7.17(6)	[3.1,5.75]	25.32/49
	5.02	60–70%	96(6)	1.28(2)	7.26(6)	[2.9,5.75]	33.46/48
	5.02	50–60%	183(8)	1.36(3)	7.34(6)	[2.9,5.75]	44.62/48
	5.02	40–50%	318(12)	1.42(3)	7.40(6)	[2.9,5.75]	63.24/48
	5.02	30–40%	512(15)	1.50(3)	7.53(6)	[2.9,5.75]	74.02/48
	5.02	20–30%	786(20)	1.55(3)	7.59(6)	[2.9,5.75]	89.96/48
	5.02	10–20%	1180(31)	1.60(3)	7.67(6)	[2.9,5.75]	101.60/48
	5.02	5–10%	1586(46)	1.62(3)	7.73(6)	[2.9,5.75]	108.88/48
	5.02	0–5%	1943(54)	1.63(3)	7.77(6)	[2.9,5.75]	113.76/48

**Table A4:** Fitting parameters of the softened-hadron formulation.

	$\sqrt{s}$ (TeV)	Class	μ	$\frac{A_{\mathrm{th}}}{A_{U}^{*}+A_{\mathrm{th}}}$	$T_{\mathrm{th}}$ (GeV)	$\sigma_{S}$ (GeV)	$q_{S}$	$\chi^{2}$/NDF
pp	13	V0M X	2.5(3)	0.28%	0.41(17)	0.33(1)	1.281(8)	4.66/43
	13	V0M IX	4.9(3)	0.45%	0.54(5)	0.37(1)	1.291(8)	1.57/43
	13	V0M VII	8.5(4)	0.76%	0.60(4)	0.39(1)	1.295(8)	6.27/43
	13	V0M VI	10.6(5)	0.93%	0.61(3)	0.40(1)	1.297(8)	9.45/43
	13	V0M IV	14.3(6)	1.21%	0.63(3)	0.41(1)	1.298(8)	14.74/43
	13	V0M III	16.7(7)	1.35%	0.64(3)	0.42(1)	1.298(8)	17.54/43
	13	V0M II	20.5(8)	1.58%	0.66(2)	0.42(1)	1.300(8)	20.68/43
	13	V0M I	26.6(11)	1.87%	0.67(2)	0.44(1)	1.300(8)	24.23/43
	13	SPD IV’	34.1(17)	1.64%	0.70(3)	0.43(1)	1.311(8)	25.58/43
	13	SPD II’	44.6(22)	1.73%	0.74(3)	0.44(1)	1.318(9)	29.49/43
	13	SPD I’	54.1(27)	1.82%	0.75(3)	0.43(1)	1.337(10)	29.77/43
pp	2.76	Min bias	4.5(2)	0.75%	0.50(5)	0.37(1)	1.280(6)	6.75/41
	5.02	Min bias	5.1(1)	0.95%	0.52(4)	0.37(1)	1.294(5)	4.36/41
	7	Min bias	5.5(1)	1.09%	0.53(4)	0.36(1)	1.301(6)	3.58/41
	8	Min bias	5.7(1)	1.19%	0.52(4)	0.36(1)	1.304(6)	2.19/41
	13	Min bias	6.4(1)	1.27%	0.54(4)	0.36(1)	1.315(5)	4.45/41
pPb	5.02	Min bias	15.9(2)	3.19%	0.53(2)	0.37(1)	1.314(7)	4.12/41
	8.16	Min bias	18.5(2)	3.42%	0.54(2)	0.36(1)	1.326(7)	4.05/41
PbPb	5.02	70–80%	45(3)	2.20%	0.49(3)	0.39(1)	1.290(4)	2.63/45
	5.02	60–70%	96(6)	3.48%	0.47(2)	0.40(1)	1.289(5)	1.71/45
	5.02	50–60%	183(8)	5.22%	0.46(2)	0.41(2)	1.289(5)	0.99/45
	5.02	40–50%	318(12)	7.29%	0.45(1)	0.41(2)	1.289(6)	0.64/45
	5.02	30–40%	512(15)	9.00%	0.45(1)	0.41(2)	1.288(6)	0.59/45
	5.02	20–30%	786(20)	10.82%	0.44(1)	0.41(2)	1.287(6)	0.87/45
	5.02	10–20%	1180(31)	12.24%	0.44(1)	0.40(2)	1.286(7)	1.54/45
	5.02	5–10%	1586(46)	13.26%	0.43(1)	0.40(2)	1.285(7)	2.13/45
	5.02	0–5%	1943(54)	14.14%	0.43(1)	0.40(2)	1.284(7)	3.25/45

**Table A5:** Fitting parameters of the two-component model.

	$\sqrt{s}$ (TeV)	Class	μ	$\frac{A_{s}}{A_{h}+A_{s}}$	$T_{s}$ (GeV)	$T_{h}$ (GeV)	*k*	$\chi^{2}$/NDF
pp	13	V0M X	2.5(3)	97.67%	0.174(14)	0.66(10)	3.26(14)	1.51/42
	13	V0M IX	4.9(3)	96.57%	0.174(11)	0.69(7)	3.16(11)	0.94/42
	13	V0M VII	8.5(4)	95.71%	0.169(11)	0.72(6)	3.09(10)	0.30/42
	13	V0M VI	10.6(5)	95.42%	0.168(11)	0.73(5)	3.06(10)	0.33/42
	13	V0M IV	14.3(6)	95.20%	0.169(11)	0.75(5)	3.05(10)	0.53/42
	13	V0M III	16.7(7)	95.13%	0.169(11)	0.77(5)	3.05(10)	0.78/42
	13	V0M II	20.5(8)	94.96%	0.170(11)	0.78(5)	3.04(10)	0.98/42
	13	V0M I	26.6(11)	94.80%	0.173(11)	0.81(5)	3.03(10)	1.41/42
	13	SPD IV’	34.1(17)	94.71%	0.165(12)	0.79(5)	2.89(10)	1.54/42
	13	SPD II’	44.6(22)	94.52%	0.165(12)	0.80(5)	2.80(9)	2.50/42
	13	SPD I’	54.1(27)	94.20%	0.163(13)	0.79(5)	2.63(9)	3.66/42
pp	2.76	Min bias	4.5(2)	97.26%	0.181(8)	0.75(6)	3.41(11)	4.70/41
	5.02	Min bias	5.1(1)	96.91%	0.177(7)	0.75(5)	3.26(10)	2.37/41
	7	Min bias	5.5(1)	96.75%	0.177(7)	0.75(5)	3.20(10)	1.48/41
	8	Min bias	5.7(1)	96.58%	0.175(9)	0.74(5)	3.17(10)	0.63/41
	13	Min bias	6.4(1)	96.51%	0.175(7)	0.76(5)	3.08(9)	2.14/41
pPb	5.02	Min bias	15.9(2)	95.72%	0.177(8)	0.81(4)	3.28(10)	1.11/41
	8.16	Min bias	18.5(2)	95.43%	0.177(8)	0.81(4)	3.16(9)	1.23/41
PbPb	5.02	70–80%	45(3)	93.85%	0.165(9)	0.67(3)	3.14(3)	6.20/56
	5.02	60–70%	96(6)	92.64%	0.163(10)	0.66(3)	3.14(3)	9.60/56
	5.02	50–60%	183(8)	91.48%	0.161(10)	0.66(2)	3.15(3)	16.42/56
	5.02	40–50%	318(12)	90.40%	0.159(10)	0.65(2)	3.15(2)	29.33/56
	5.02	30–40%	512(15)	89.96%	0.160(10)	0.66(2)	3.19(2)	40.94/56
	5.02	20–30%	786(20)	89.33%	0.159(10)	0.66(2)	3.20(2)	56.72/56
	5.02	10–20%	1180(31)	89.03%	0.160(11)	0.67(2)	3.23(2)	70.12/56
	5.02	5–10%	1586(46)	88.77%	0.158(11)	0.66(2)	3.25(2)	81.10/56
	5.02	0–5%	1943(54)	88.56%	0.158(11)	0.66(2)	3.26(2)	90.61/56
